# The role of sigmoidoscopy in thediagnosis and treatment of sigmoid volvulus

**DOI:** 10.12669/pjms.321.8410

**Published:** 2016

**Authors:** Sabri Selcuk Atamanalp, Refik Selim Atamanalp

**Affiliations:** 1 Prof. Sabri Selcuk Atamanalp, MD, Department of General Surgery, Faculty of Medicine, Ataturk University, 25040, Erzurum, Turkey; 2 Refik Selim Atamanalp, English Medicine Program, Faculty of Medicine, Ataturk University, 25040, Erzurum, Turkey

**Keywords:** Sigmoid colon, Volvulus, Sigmoidoscopy, Diagnosis, Treatment

## Abstract

Sigmoid volvulus (SV) is a rare form of acute intestinal obstruction in which the sigmoid colon wraps around itself. The disease generally presents as a mechanical bowel obstruction with clinical features that are not pathognomonic. Similarly, X-ray films are not diagnostic in most cases. It is difficult to establish the correct preoperative diagnosis when CT and MRI are not used.

The principal strategy in the treatment of SV in uncomplicated patients is emergency endoscopic detorsion followed by elective surgery; emergent surgery is required in patients with bowel gangrene, bowel perforation, peritonitis, or unsuccessful endoscopic treatment.

In this review, we have discussed the role of sigmoidoscopy in the diagnosis and treatment of SV. Additionally, we have retrospectively and prospectively evaluated our 49-year, 987-patient clinical experience, the largest single-center SV series ever reported.

## INTRODUCTION

First described by von Rokitansky in 1836, sigmoid volvulus (SV) refers to the wrapping of the sigmoid colon around its own base.^1^-^3^ The incidence of SV is relatively high in South Africa, the Middle East, Northern Europe, Latin America and Australia,^1^,^3^-^6^ but the incidence in the United states is 1.67 per 100 000 person-years.^5^ SV most commonly occurs in adult men and usually presents in the 4^th^ to 8^th^ decades of life; the male to female ratio ranges from 1.4/1 to 4/1.^1^,^3^,^7^ The presence of an elongated sigmoid colon with a narrow mesentery (dolichosigmoid) is a prerequisite for SV, which is thought to be related to advanced age, male gender, high altitude, dietary or defecation habits, and some pathologies such as megacolon.^1^,^8^

SV generally presents as an acute large bowel obstruction. The most common clinical features include abdominal pain, abdominal distention, and obstipation, which are known as the classical SV triad.^1^,^3^,^4^,^9^ It may be difficult to make an accurate preoperative diagnosis of SV without using sigmoidoscopy, CT, or MRI; the diagnosis is currently made under laparotomy or on autopsy in 10-15% of patients.^1^-^3^,^7^,^10^

The principal strategy in the treatment of SV in uncomplicated patients is emergency endoscopic detorsion followed by elective surgery, while emergency surgical treatment is needed in patients with bowel gangrene, bowel perforation, peritonitis, or unsuccessful endoscopic detorsion. The mean morbidity rate of SV is 12.5%, while the mortality rate ranges from 8 to 28.3%.^1^,^11^-^13^

### Indications and contraindications of sigmoidoscopy

Any evidence or suspicion of a large bowel obstruction, particularly SV, is one of the main indications of diagnostic and/or therapeutic sigmoidoscopy.^1^,^3^,^13^ Abdominal pain, abdominal distention, and obstipation (which are the symptoms of the classical SV triad) are observed on average in 93%, 89.9%, and 83% of SV patients, respectively.^1^-^4^,^6^,^14^,^15^ Abdominal X-ray film shows SV findings, including a sigmoid dilatation with intestinal air-fluid levels, in 57-90% of patients.^1^,^3^,^10^,^16^ CT or MR is able to accurately identify SV in 96.6% and 97.4% of patients, respectively. Signs that can help diagnose SV on CT or MR include the whirl sign in the sigmoid mesentery, sigmoid dilatation, and intestinal air-fluid levels.^1^,^3^,^10^,^17^ Contrast enema has been used to diagnose SV in the past (mostly in children) but has a 66.7-78.6% success rate and a mortality rate that ranges from 7.7-9%.^2^,^18^ Contrast enema is not advised because of the possibility of bowel perforation, peritonitis, and risk of missing bowel gangrene.^1^,^3^,^13^ Sigmoidoscopy helps to diagnose the bowel torsion, shows the viability of the bowel mucosa, and contributes to the bowel detorsion. Therefore, rigid or (preferably) flexible sigmoidoscopy is one of the best methods of diagnosing SV and is the preferred initial treatment of SV.^1^,^3^,^13^,^19^

One of the main contraindications to sigmoidoscopy is evidence or suspicion of bowel gangrene, bowel perforation, or peritonitis, which may clinically manifest as melanotic stool during anamnesis or rectal examination, guarding/rigidity, or rebound tenderness.^1^,^3^,^13^ Sigmoid gangrene develops in 6.1-30.2% of all SV cases and in 10.7-93.4% of surgically treated SV cases. Melanotic rectal stool is observed in 7.3-11.8% of SV patients, while bowel gangrene is diagnosed on average in 5.5% of patients during sigmoidoscopy, causing the need to terminate the procedure. Guarding/rigidity or rebound tenderness is found in 8.9-14.9% of SV patients.^1^,^3^,^4^,^13^,^14^,^20^,^21^

### Sigmoidoscopy in diagnosis

**Fig.1:**
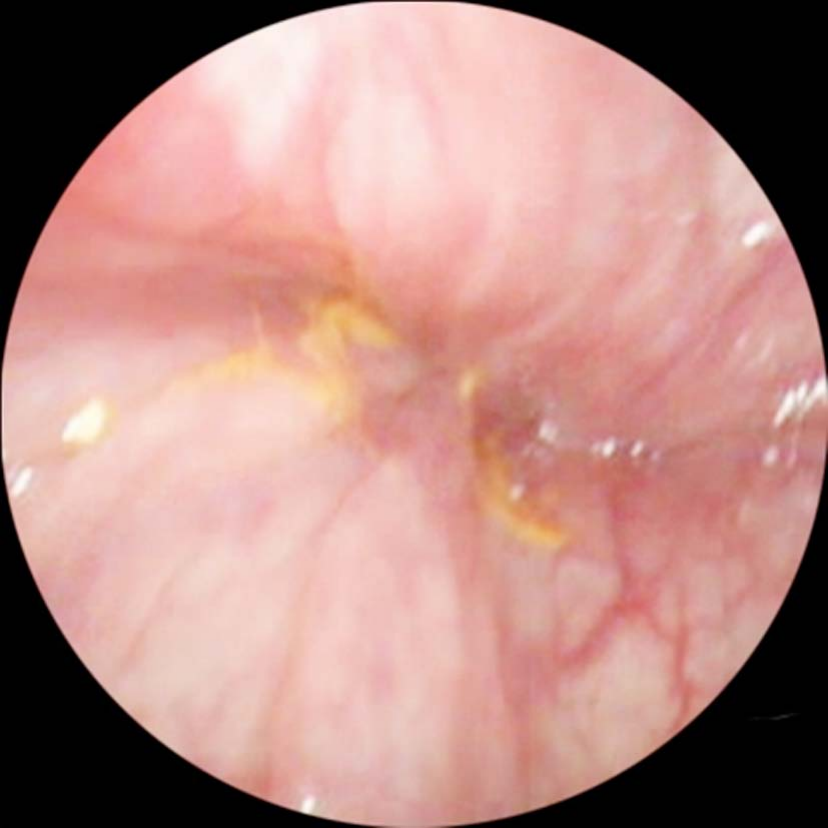
Endoscopic view of the sigmoid colon in a patient with sigmoid volvulus (spiral sphincter-like twist of the obstructed bowel lumen)

Sigmoidoscopy helps establish the diagnosis of SV. The classical finding on sigmoidoscopy is a spiral sphincter-like twist of the lumen, usually 20-30 cm from the anal verge (Fig.1); additionally, the inability to insert the endoscope into the proximally twisted site helps lead to the correct diagnosis. Sigmoidoscopy allows for direct visualization of the bowel mucosa viability and may also be used in the differential diagnosis of SV by identifying the other causes of bowel obstruction, such as bowel malignancies or megacolon.^1^,^3^,^5^,^6^,^10^,^14^,^19^,^22^-^25^

Although sigmoidoscopy is thought to have a high diagnostic value in patients with SV (with a 76-100% diagnostic success rate), there is no quantitative data available in the literature commenting on the overall diagnostic role of sigmoidoscopy.^1^,^3^,^10^,^14^,^19^,^22^-^25^

### Sigmoidoscopy in treatment

SV requires an emergency treatment following an early and effective resuscitation.^1^,^12^,^13^ Endoscopic detorsion, including gentle inserting of the endoscope with rotation of the tip of the instrument towards the opposite side of the torsion direction while providing minimal air insufflation, is the initial treatment of choice in uncomplicated SV patients. A 48.1-100.0% success rate, 0.0-26.4% morbidity rate, and 0.0-19.0% of mortality rate has been reported using this technique.^1^-^7^,^9^,^10^,^12^-^15^,^18^,^19^,^21^-^33^ The results of endoscopic treatments in various series are shown in Table-I.

**Table-I:** Endoscopic treatments of patients with sigmoid volvulus in different series.

Author	Years	Patient No.	Success (%)	Morbidity (%)	Mortality (%)	Follow-up	Recurrence (%)
String and DeCosse^22^	1971	17	64.7		9.1		20.0
Arnold and Nance^2^	1973	114	76.3	0.9	9.0	2 years	55.5
Ballantyne et al^4^	1985	31	83.9			49 months	19.4
Arigbabu et al^26^	1985	92	88.2				
Bak and Boley^15^	1986	43	90.7	4.7	2.3		
Brothers et al^19^	1987	29	55.2		8.0		57.0
Oncu et al^27^	1991	18	55.6		0.0		
Grossmann et al^14^	2000	189	81.5				69.7
Salas et al^18^	2000	28	60.7				
Turan et al^28^	2004	81	48.1	3.7	0.0		15.0
Bhuiyan et al^29^	2005	17	58.8		5.9		
Oren et al^12^	2007	562	78.3	2.5	0.7	Early	3.2
Safioleas et al^21^	2007	33	78.8		3.0	14 months	41.7
Heis et al^7^	2008	25	68.0		0.0		
Jangjoo et al^23^	2010	75	89.7				
Mulas et al^24^	2010	24	70.1	26.4			
Tan et al^25^	2010	29	82.8	3.4			
Swenson et al^9^	2012	28	78.6		19.0	106 days	47.6
Lou et al^30^	2013	28	92.9	0.0	0.0		26.9
Yassaie et al^31^	2013	31	100.0		0.0	31 days	61.3
Atamanalp^13^	2013	673	77.3	2.1	0.6	Early	4.4
Maddah et al^32^	2014	80	100.0				12.9
Sugimoto et al^33^	2014	71	100.0	0.0	0.0	200 days	55.6

During endoscopic detorsion, a variety of anesthetics or sedatives may be used.^1^,^3^,^13^ In detorsioned cases, a rectal tube may be placed in the sigmoid colon to prevent an early recurrence; it is withdrawn following a radiograph, which is obtained a few hours later.^1^,^3^,^12^,^13^,^23^-^25^,^27^,^31^,^32^ The use of flexible endoscopes instead of rigid endoscopes may increase the success rate and decrease the complication and mortality rates; overall, flexible endoscopes are better tolerated by the patients.^1^,^3^,^22^-^26^,^28^ Similarly, the success rate may be increased by the use of colonoscopes instead of sigmoidoscopies.^1^,^3^,^13^,^22^,^23^,^28^

The main complications of sigmoidoscopy-treated SV as well as the most common causes of sigmoidoscopy-related deaths are bowel perforation, peritonitis, shock, fluid-electrolyte imbalances, renal insufficiency, and cardiopulmonary problems.^1^,^3^,^12^,^13^,^25^,^28^ Because SV has a tendency to recur after endoscopic detorsion (3.2-69.7% of successfully detorsioned SV cases),^2^,^4^,^10^,^12^-^14^,^19^,^21^-^23^,^28^,^30^-^34^ elective surgery is recommended after 2-3 days in a select group of patients;^1^,^3^,^12^,^13^,^23^,^34^,^35^ this recommendation is particularly applicable to ASA 1-3 patients, in whom perioperative mortality is minimal.^34^

## SIGMOIDOSCOPY IN SPECIAL SITUATIONS

### Sigmoidoscopy in Childhood

SV is extremely rare in childhood, with less than 100 cases reported in the literature.^1^,^3^,^13^,^18^,^32^,^36^,^37^ In spite of the fulminant clinical presentation, it is difficult to obtain a preoperative accurate diagnosis.^36^,^37^ Although hydrostatic reduction via barium, water-soluble contrast, or saline was previously used during non-operative treatment, endoscopic reduction performed via pediatric endoscopes may also be used successfully in uncomplicated patients.^1^,^18^,^36^,^37^ The morbidity of SV in childhood remains high and occurs in approximately 30% of all patients; the mortality is also startling, which is observed more than 25% of patients.^18^,^36^,^37^

### Sigmoidoscopy in the elderly

SV is common in the elderly and approximately 50% of SV patients are over 60 years old.^1^,^5^,^13^,^15^,^21^,^38^ Abnormal defecation and chronic constipation, which are features normally found in the elderly, may cloud the clinical picture. The clinical picture in these patients may therefore be less diagnostic.^1^,^3^,^5^,^13^,^15^,^21^,^38^ Endoscopic reduction is the first choice in the treatment of uncomplicated patients, and the avoidance of emergency surgery improves the prognosis.^1^,^5^ These elderly patientssuffer from high morbidity, which occurs in 6-24% of cases. Notably, the mortality increases to 75% after the age of 70; 50-85% of these patients have serious comorbidities.^1^,^5^,^15^,^21^,^38^

### Sigmoidoscopy during Pregnancy

SV is relatively rare in pregnancy. As of 2014, there were fewer than 100 cases reported in the literature.^1^,^3^,^13^,^39^,^40^ Abdominal pain, nausea, and vomiting are normal findings in pregnancy; as such, these clinical findings are not reliable diagnostic features of SV.^1^,^3^,^39^,^40^ The management of SV in pregnancy requires a multidisciplinary approach involving general surgery, obstetrics, and neonatology.^39^,^40^ Although endoscopic detorsion was thought to be unsuccessful in most pregnant patients in the past due to an enlarged uterus as a mechanical impediment,^40^ gentle flexible endoscopic detorsion under careful monitoring is recommended as treatment of choice in all trimesters of pregnancy in the treatment of uncomplicated patients, but is particularly true for those women in the first and second trimesters.^39^ SV has a poor prognosis in pregnancy, with reported 6-60% maternal and 20-50% fetal mortality rates.^1^,^39^,^40^

### Clinical Experience

The incidence of SV is high in Turkey, particularly in Eastern Anatolia^3^,^13^ where our university clinic is located. To the best of our knowledge, this report represents the largest single-center SV series.

A total of 987 patients with SV were treated over a 49-year period between June 1966 and June 2015 in the Department of General Surgery, Faculty of Medicine, Ataturk University. The data were collected retrospectively till 1986, and prospectively after. After resuscitation and clinical examination, abdominal X-rays were obtained for all patients (although CTs or MRIs have been obtained in several stable patients in recent years). Emergency surgery was performed in patients with acute abdominal findings, melanotic stool, and unsuccessful non-operative detorsion. Sigmoidoscopy was used in the diagnosis of several stable patients but has been used in the treatment of all stable SV patients. We used rigid sigmoidoscopy in the early years but have tended to use flexible sigmoidoscopy or colonoscopy over the past 26 years. In successfully detorsioned patients, a rectal tube was inserted into the sigmoid colon and was left in place for 12-24 hours. Elective surgery was recommended in several stable patients.

Diagnostic sigmoidoscopy was used in 151 patients; the correct diagnosis was obtained in 149 of those patients (accuracy rate, 98.7%). Endoscopic misdiagnosis included colonic invagination in one patient and partial colonic volvulus in another; notably, there were colonic malignancies in both patients. Nonoperative therapeutic procedures were used in 712 patients; barium enema in 13 patients, rigid sigmoidoscopy in 351 patients, and flexible sigmoidoscopy in 348 patients. The results of these procedures are shown in table 2. When the patients with bowel gangrene are excluded, the therapeutic success rate of then on operative procedures is 82.1%, with a highest success rate in the flexible sigmoidoscopy group (82.9%). In the nonoperatively treated group, 5 patients (0.7%) died: 3 died from toxic shock and two died from peritonitis; the lowest mortality rate was in the flexible sigmoidoscopy group (0.3%). Complications were observed in 17 of the nonoperatively treated patients (2.4%). These complications included renal insufficiency in 13 patients, myocardial infarction in two patients, and peritonitis in 2 patients, with the lowest morbidity rate in the flexible sigmoidoscopy group (1.4%). Early recurrence was observed in 26 patients (4.7%) with the lowest early recurrence rate in the rigid sigmoidoscopy group (3.3%).

**Table-II:** Non-operative procedures in patients with sigmoid volvulus and their respective results.

	Barium enema	Rigid sigmoidoscopy	Flexible sigmoidoscopy	Total
Total	13(1.8%)	351(49.3%)	348(48.9%)	712
Success	9(69.2%)	274(78.1%)	266(76.4%)	549(77.1%)
Failure	4(30.8%)	61(17.4%)	55(15.8%)	120(16.9%)
Bowel gangrene	0(0.0%)	16(4.6%)	27(7.8%)	43(6.0%)
Success except gangrenous cases	9/13(69.2%)	274/335(81.8%)	266/321(82.9%)	549/669(82.1%)
Mortality	1(7.7%)	3(0.9%)	1(0.3%)	5(0.7%)
Morbidity	3(23.1%)	9(2.6%)	5(1.4%)	17(2.4%)
Earlyr ecurrence (in the hospitalization period)	1(11.1%)	9(3.3%)	16(6.0%)	26(4.7%)

## DISCUSSION

As regards the diagnosis of SV, clinical features are not pathognomonic, and abdominal X-ray films are usually not helpful. However, CT and MR are almost always diagnostic. Rigid or (preferably) flexible sigmoidoscopy helps to the diagnosis of SV by direct visualization of the obstructive bowel lumen. Additionally, sigmoidoscopy may demonstrate the viability of the bowel mucosa and identify other potential causes of bowel obstruction.

Spontaneous detorsion of SV is not common and therefore requires emergency treatment. Rigid or (preferably) flexible endoscopic detorsion is the initial treatment of choice in SV in the absence of bowel gangrene, bowel perforation, or peritonitis. Hydrostatic reduction has historical value, and endoscopic detorsion via pediatric endoscopes is the preferable treatment method in children. Endoscopic reduction is the treatment of choice in the elderly, improving the overall prognosis by avoiding emergent surgery. Endoscopic detorsion via monitorization is also the first choice in pregnancy, particularly in the first and second trimesters.

There is minimal morbidity and mortality from flexible sigmoidoscopy-treated SV. Because SV has a tendency to recur and because each subsequent SV episode has different morbidity and mortality, elective surgery is recommended in a select group of patients.
